# Supplementary material for: The role of training on smallholder farmers’ adoption of orange-fleshed sweet potato in Ethiopia
Source: PLoS One. 2026 Jan 20;21(1):e0340709. doi: 10.1371/journal.pone.0340709 (PMC12818620; doi:10.1371/journal.pone.0340709)
Supplement: S1 Table — (DOCX) [file pone.0340709.s001.docx]

**Table 1A Farmers’ socio-demographics and farm characteristics, for both OFSP adopters (n=35) and non-adopters (n=30)**

| **Variable** | **OFSP- adopters**  **n** | **Non-OFSP adopters**  **n** |
| --- | --- | --- |
| **Gender** | | |
| Male | 26 | 24 |
| Female | 9 | 6 |
| **Marital status** | | |
| Married | 33 | 29 |
| Unmarried | 2 | 1 |
| **Variable** | **Mean** | **mean** |
| **Level of education** | | |
| Number of years in school | 5.3 | 5.0 |
| **Extension Service visit** | | |
| (1= Never, to 5=Everyday) | 3 | 3 |
| **Farm size** | | |
| Total land size (hectares) | 0.51 | 0.54 |
| **Annual household income satisfaction** | | |
| (1= very unsatisfied, to 5=very satisfied) | 3.7 | 3.5 |
| **Age** ( in years) | 41.0 | 40.4 |
| **Farming experience** (in years) | 25.0 | 28.1 |
| **Number of children** (below 5 yrs. old) | 1.9 | 2.31 |
| **Membership farmers cooperatives in years** | 13.16 | 11.4 |
