# Supplementary material for: The role of training on smallholder farmers’ adoption of orange-fleshed sweet potato in Ethiopia
Source: PLoS One. 2026 Jan 20;21(1):e0340709. doi: 10.1371/journal.pone.0340709 (PMC12818620; doi:10.1371/journal.pone.0340709)
Supplement: S2 Table — (DOCX) [file pone.0340709.s002.docx]

**Table 2A. TAM and TPB model results, pre & post-treatments (n=65)**

| **TAM Model** | **# trained farmers** | **TAM Model** | **# Non-trained**  **farmers** |
| --- | --- | --- | --- |
| ***Perceived factors that made cultivating OFSP easy by non-OFSP adopters (Perceived easiness) (PE)* (pre-treatment)** |  | ***Perceived factors that made cultivating OFSP easy by OFSP adopters (Perceived easiness) (PE) (*pre-treatment)** |  |
| Suitable or fertile land | 8 | Lower labor requirement | 6 |
| Access to irrigation facilities | 6 | Familiar cooking methods with other root crops | 5 |
| Minimal water use during cooking | 6 | Minimal soil or land preparation | 5 |
| Natural suitability for cultivation across environments | 3 | Similar land & cooking to WFSP | 4 |
| Nutritional benefits & market potential | 3 | Early maturity nature | 4 |
| Access to land | 2 | Minimal water requirement during cultivation | 4 |
| Availability of agricultural tools | 1 | High yield per unit of land | 3 |
|  |  | Resilience and adaptability to various environmental conditions | 2 |
|  |  | Compatibility with existing farming practices | 2 |
|  |  | Experience with cultivating and harvesting OFSP | 1 |
| ***Perceived factors that made cultivating OFSP easy by non-OFSP adopters (Perceived easiness) (PE) (Trained)*** |  | ***Perceived factors that made cultivating OFSP easy by non-OFSP adopters (Perceived easiness) (PE) (Not- trained)*** |  |
| Access to free vines from your side | 7 | Access to free vines from your side | 6 |
| Trust in the source of the vines | 3 | Cultural acceptance | 4 |
| Access to irrigation | 3 | Access to land | 2 |
| Willingness to try new varieties (technology) | 1 |  |  |
| Similar cooking process with WFSP | 1 |  |  |
| Access to land | 1 |  |  |
| ***Perceived factors that made cultivating OFSP easy by OFSP adopters (Perceived easiness) (PE) (Trained)*** |  | ***Perceived factors that made cultivating OFSP easy by OFSP adopters (Perceived easiness) (PE) (Not-trained)*** |  |
| Resilience to certain environmental conditions | 9 | Cost-effectiveness | 8 |
| Straight forward/ uncomplicated agricultural practices/multiplication | 8 | Early maturing nature helps to fill food gap | 4 |
| Availability of training from your side | 6 | My knowledge about OFSP’s high nutritional benefit | 4 |
|  |  | Compatibility with existing farming practices (inter-crop) | 3 |
| ***Perceived factors that made cultivating OFSP challenging by non-OFSP adopters (Perceived challenges) (PC) (pre-treatment)*** |  | ***Perceived factors that made cultivating OFSP challenging by OFSP adopters (Perceived challenges) (PC) (pre-treatment)*** |  |
| Limited access to vines | 21 | Limited access to quality vines | 11 |
| Lack of sufficient knowledge & awareness | 8 | Space requirement & land scarcity | 9 |
| Pest infestations & management | 7 | Water scarcity & irrigation challenges | 6 |
| Consumer suspicion & market dynamics | 2 | Knowledge gaps in cultivation techniques | 4 |
| Financial constraint | 1 | Competition for land with cash crops | 1 |
| Lack of local role model | 1 | Logistic challenges in market supply | 1 |
|  |  | Climate change impact on crop yield | 1 |
|  |  | Policy & institutional support | 1 |
|  |  | Lack of effective training & support program | 1 |
|  |  | Storage & field management practices | 1 |
| ***Perceived factors that made cultivating OFSP challenging by non-OFSP adopters (Perceived challenges) (PC) (Trained)*** |  | ***Perceived factors that made cultivating OFSP challenging by non-OFSP adopters (Perceived challenges) (PC) (Not-trained)*** |  |
| Lack of planting materials | 9 | Lack of technical knowledge (was not selected for the training) | 6 |
| Water scarcity/ lack of irrigation | 4 | Lack of planting materials | 3 |
| Lack of sufficient knowledge about the crop | 3 | Fear of trying new things | 2 |
|  |  | Labor requirement | 1 |
|  |  | Market acceptance (consumer are suspicious) | 1 |
| ***Perceived factors that made cultivating OFSP challenging by OFSP adopters (Perceived challenges) (PC) (Trained)*** |  | ***Perceived factors that made cultivating OFSP challenging by OFSP adopters (Perceived challenges) (PC) (Not-trained)*** |  |
| Shortage of land for large production | 10 | Lack of technical knowledge (was not selected for the training) | 8 |
| Most of the vines were dry when they arrive | 7 | Most of the vines were dry when they arrive | 6 |
|  |  | The vines have short life (must be planted quickly) | 2 |
|  |  | Lack of technical support from experts | 1 |
|  |  | Availability of pests &rodents | 1 |
|  |  | Lack of recurring training | 1 |
| ***Perceived usefulness of OFSP by non-OFSP adopters (PU) (pre-treatment)*** |  | ***Perceived usefulness of OFSP-by-OFSP adopters (PU) (pre-treatment)*** |  |
| Nutritional content | 9 | Nutritional content | 15 |
| High yield potential | 8 | High yield potential | 9 |
| Color | 5 | Early maturity | 5 |
| Taste | 4 | Cost-effective | 4 |
| Consumer appeal | 1 | Marketability or market competitiveness | 1 |
| Economic viability | 1 | Pest & disease resistance | 1 |
| Marketability or market competitiveness | 1 | Drought resistance | 1 |
| ***Perceived usefulness of OFSP by non-OFSP adopters (PU) (Trained)*** |  | ***Perceived usefulness of OFSP by non-OFSP adopters (PU) (Not-trained)*** |  |
| Nutritional value | 9 | Nutritional value | 9 |
| Good taste | 3 | Color | 3 |
| Early maturity | 3 | Size of the tuber | 1 |
| Size of the tuber | 1 |  |  |
| ***Perceived usefulness of OFSP by OFSP adopters (PU) (Trained)*** |  | ***Perceived usefulness of OFSP-by-OFSP adopters (PU) (Not-trained)*** |  |
| High yield | 11 | High yield | 8 |
| Nutritional value | 4 | Nutritional value | 6 |
| Economic opportunity | 3 | Cost-effectiveness | 3 |
|  |  | Dual purpose (human &livestock) | 2 |
| ***Perceived opportunities by non-OFSP adopters (pre-treatment)*** |  | ***Perceived opportunities by OFSP adopters (pre-treatment)*** |  |
| Suitable agro- ecology | 10 | Availability of water | 19 |
| Soil fertility or available land | 8 | Nutritional benefit | 6 |
| Extension service support | 6 | The need for dietary diversification | 7 |
| High market demand for the crop | 3 | Availability of information about the crop | 2 |
| Fertilizer availability | 2 | Agronomic benefits | 2 |
|  |  | Farming experience | 1 |
| ***Perceived opportunities by non-OFSP adopters (Trained)*** |  | ***Perceived opportunities by non-OFSP adopters (Not-trained)*** |  |
| Nutritional benefits | 9 | Availability of health &food security issues | 9 |
| Market demand | 6 | Availability of land | 4 |
| Free vines | 1 |  |  |
| ***Perceived opportunities by OFSP adopters (Trained)*** |  | ***Perceived opportunities by OFSP adopters (Not-trained)*** |  |
| Agronomic benefits | 9 | Agronomic benefits | 8 |
| Economic benefits | 6 | Need for dietary diversification | 6 |
| Access to information /Training | 3 | Water/ irrigation | 5 |
| Sweet potato being staple crops | 1 |  |  |
| ***Perceived barriers by non-OFSP adopters (pre-treatment)*** |  | ***Perceived barriers by OFSP adopters (pre-treatment)*** |  |
| Knowledge gaps | 15 | Access to vines | 12 |
| Access to vines | 8 | Knowledge gaps | 12 |
| Lack of government support | 2 | Land scarcity | 2 |
| Cultural perceptions for new crops | 1 | Climate change impacts | 1 |
| Lack of risk taking behaviors | 1 | Water unavailability (irrigation cost is high) | 1 |
| Lack of pesticides & herbicides | 1 |  |  |
| Lack of market access | 1 |  |  |
| ***Perceived barriers by non-OFSP adopters (Trained)*** |  | ***Perceived barriers by non-OFSP adopters (Not-trained)*** |  |
| Access to timely and quality vines | 8 | Lack of technical knowledge on cultivation | 7 |
| Limited land | 5 | Lack of awareness about the crop | 6 |
| Financial challenges | 3 |  |  |
| ***Perceived barriers by OFSP adopters (Trained)*** |  | ***Perceived barriers by OFSP adopters (Not-trained)*** |  |
| Water scarcity /irrigation is challenges | 11 | Climate change | 7 |
| Infrastructure challenge | 6 | Consumer fear of trying new varieties | 6 |
|  |  | Limited extension service | 6 |
| **TPB model** |  | **TPB model** |  |
| ***Attitude toward behavior non-OFSP adopters (pre-treatment)*** |  | ***Attitude toward behavior OFSP adopters (pre-treatment)*** |  |
| Experience of previous adopters | 29 | Nutritional benefits | 21 |
|  |  | High yield | 15 |
| ***Attitude toward behavior non-OFSP adopters (Trained)*** |  | ***Attitude toward behavior OFSP adopters (Trained)*** |  |
| Early maturity | 10 | Ease of cultivation | 10 |
| Adaptability of the crop in the agroecology | 6 | Early maturity | 7 |
| ***Attitude toward behavior non-OFSP adopters (Not-trained)*** |  | ***Attitude toward Behavior OFSP adopters (Not-trained)*** |  |
| Nutritional benefits | 7 | Nutritional benefits | 10 |
| High yield | 6 | High yield | 9 |
| ***Strategies suggested by non-OFSP adopters to improve issues related to the barriers of adoption & continuous adoption (pre-treatment)*** |  | ***Strategies suggested by non-OFSP adopters to improve issues related to the barriers of adoption & continuous adoption (pre-treatment)*** |  |
| Building strong value chains | 8 | Comprehensive training programs | 11 |
| Demonstration plots & field days | 6 | Creating awareness campaigns | 8 |
| Peer learning networks | 5 | Policy advocacy & institutional support | 5 |
| Technical support & capacity building | 4 | Experience sharing among farmers | 4 |
| Market development & demand creation | 2 | Continuous monitoring & evaluation | 4 |
| Infrastructure development | 1 | Ensuring access to quality vines | 3 |
| Model farmers program | 1 | Strategic partnerships among stakeholders | 2 |
| Using risk mitigation strategies | 1 | Cooperative learning & collaboration | 1 |
| Promoting cluster farming | 1 | Consultation centers | 1 |
| ***Strategies suggested by non-OFSP adopters to improve issues related to the barriers of adoption & continuous adoption (Trained)*** |  | ***Strategies suggested by non-OFSP adopters to improve issues related to the barriers of adoption & continuous adoption (Not-trained)*** |  |
| Financial support or incentives | 7 | Farm demonstration program & farm visits | 7 |
| Market development & strengthening linkages | 4 | Comprehensive awareness creation campaigns | 5 |
| Research & development investments | 4 | Cluster farming | 1 |
| Vine multiplication programs on selected farms | 1 |  |  |
| ***Strategies suggested by OFSP adopters to improve issues related to the barriers of adoption & continuous adoption (Trained)*** |  | ***Strategies suggested by OFSP adopters to improve issues related to the barriers of adoption & continuous adoption (Not-trained)*** |  |
| Recurring trainings and workshops | 9 | Community engagement & peer-peer learning | 8 |
| Establishment of vine distributions centers | 4 | Fostering stakeholders’ partnerships | 6 |
| Continues monitoring & evaluation | 3 | Policy support & advocacy | 3 |
| Utilizing digital platforms to disseminate information | 1 | Providing training for extension service providers | 2 |
| ***Behavioral intention (BI) (factors affecting future adoption for non-OFSP adopters (pre-treatment)*** |  | ***Behavioral intention (BI) (factors affecting future adoption for OFSP adopters (pre-treatment)*** |  |
| Market demand & stability | 5 | Knowledge | 6 |
| Health benefits | 3 | Risk tolerance | 5 |
| Technical assistance | 3 | Financial considerations | 5 |
| Agronomic benefits | 3 | Previous experience with OFSP | 4 |
| Cost-effectiveness | 3 | Access to continuous information & training | 4 |
| Knowledge & education | 3 | Planting material | 4 |
| Success stories | 3 | Nutritional value | 3 |
| Availability of quality planting materials | 2 | Technical support | 3 |
| Support (including policy) & training | 2 | Yield | 2 |
| Community & cooperative support | 1 | Cost- effectiveness | 2 |
| Climate resilience | 1 | Individual behavioral | 1 |
| Incentives for best adopters | 1 | Cultural factors | 1 |
| Access to credit | 1 | Age | 1 |
| Color | 1 | Taste | 1 |
| Nutritional awareness | 1 | Shifting priorities | 1 |
| Good taste | 1 |  |  |
| ***Behavioral intention (BI) (factors affecting future adoption for non-OFSP adopters (Trained)*** |  | ***Behavioral intention (BI) (factors affecting future adoption for non-OFSP adopters (Not-trained)*** |  |
| Adaptability & suitability to agro-ecology & climatic conditions | 9 | Success stories | 7 |
| Access to credit | 5 | Trainings & information from extension workers | 5 |
| Ease of cultivation | 2 | Taste | 1 |
| ***Behavioral intention (BI) (factors affecting future adoption for OFSP adopters (Trained)*** |  | ***Behavioral intention (BI) (factors affecting future adoption for OFSP adopters (Not-trained)*** |  |
| Previous yield data | 9 | Market demand & profitability | 9 |
| Nutritional value | 5 | Success stories | 5 |
| Previous experience with the crop | 4 | Access to quality planting material | 3 |
| Benefits for my livestock | 3 | Feedback from my family | 2 |
| **Subjective norm** |  |  |  |
| ***Perceived factors that made cultivating OFSP important by other farmers in the village (pre-treatment) (Non-OFSP adopters)*** |  | ***Perceived factors that made cultivating OFSP challenging by other farmers in the village(pre-treatment) (OFSP adopters)*** |  |
| Nutritional benefits (vitamin A content) | 14 | High initial investment costs | 15 |
| Potential for higher income (market demand) & yield | 9 | Access to information | 9 |
| Agronomic benefits (resilience to pests & diseases & drought) | 6 | Land scarcity or quality issues | 9 |
|  |  | Uncertainty about cultural perceptions & acceptance | 3 |
| ***Perceived factors that made cultivating OFSP important by other farmers in the village (Non-adopters& trained)*** |  | ***Perceived factors that made cultivating OFSP challenging by other farmers in the village (non-OFSP adopters & not-trained)*** |  |
| Nutritional benefits | 9 | Pest and disease management (specifically worms) | 9 |
| Potential for higher income (market demand & yield) | 5 | Access to vines | 5 |
| Agronomic benefits (resilience to pests and disease & drought) | 2 | Market acceptance & demand | 3 |
| ***Perceived factors that made cultivating OFSP important by other farmers in the village (OFSP-adopters& trained)*** |  | ***Perceived factors that made cultivating OFSP challenging by other farmers in the village (OFSP adopters & Not-trained)*** |  |
| Enhanced consumer appeal | 7 | Knowledge & information gap | 19 |
| Ease of cultivation (low input requirement) | 6 |  |  |
