# Supplementary material for: The role of training on smallholder farmers’ adoption of orange-fleshed sweet potato in Ethiopia
Source: PLoS One. 2026 Jan 20;21(1):e0340709. doi: 10.1371/journal.pone.0340709 (PMC12818620; doi:10.1371/journal.pone.0340709)
Supplement: Attachment — Submitted filename: Plos One Rebuttal Letter.pdf [file pone.0340709.s006.pdf]

Rebuttal letter: ID PONE-D-25-37573

Dear Dr. Julius Adewopo

First and foremost, we would like to thank you and the reviewers for the comments and suggestions. We are grateful that the editorial and the reviewer staff have appreciated our study and allowed correction of our manuscript (ID PONE-D-25-37573).

Below, we present our responses to all questions and suggestions. We have introduced corrections where we found it justified and possible. The corrected fragments of text have been highlighted in yellow.

Sincerely

**Editor**	**Response**
Please ensure that your manuscript meets PLOS ONE's style requirements, including those for file naming.	Thank you for this comment.<br><br>As suggested, all files have now been renamed clearly according to journal standards, and the manuscript has been formatted to align with the journal's structure and submission guidelines.
In the ethics statement in the Methods, you have specified that verbal consent was obtained. Please provide additional details regarding how this consent was documented and witnessed, and state whether this was approved by the IRB	We appreciate the editor's observation.<br><br>We have now added details explaining how verbal consent was obtained, documented, and witnessed, and confirmed that this process was explicitly approved by the Research Ethics and Integrity Committee of the Africa Centre of Excellence (ACE) for Climate Smart Agriculture and Biodiversity Conservation (Ref. No. AGE 01/11/2024). The revised statement clarifies compliance with ethical standards for informed consent procedures.<br><br>…" Given that many participants were smallholder farmers with limited literacy, the Committee approved verbal informed consent. Prior to each interview, the study objectives, voluntary participation, and confidentiality assurances were clearly explained in the local language. Each participant's verbal consent was recorded in the enumerators' field log and witnessed by our trained field assistant. This procedure was reviewed and approved by the Ethics Committee as part of the IRB protocol.2.4 Data analysis".

Thank you for stating the following financial disclosure:<br><br>"Ghent University Special Research Fund (BOF-01W00421)"<br><br>Please state what role the funders took in the study. If the funders had no role, please state: "The funders had no role in study design, data collection and analysis, decision to publish, or preparation of the manuscript.<br>If this statement is not correct, you must amend it as needed.<br>Please include this amended Role of Funder statement in your cover letter; we will change the online submission form on your behalf.	Thank you for your feedback.<br><br>We have included the following amended Role of Funder statement in the cover letter as requested:<br>"Ghent University Special Research Fund (BOF-01W00421) supported the doctoral studies of the first author, but the funders had no role in study design, data collection and analysis, decision to publish, or preparation of the manuscript."<br><br>Please let us know if further clarification is needed. We appreciate your guidance to ensure compliance with PLOS ONE's funding disclosure policies.
Please note that funding information should not appear in the Acknowledgments section or other areas of your manuscript. We will only publish funding information present in the Funding Statement section of the online submission form. Please remove any funding-related text from the manuscript.	Thank you for the clarification.<br><br>In accordance with PLOS ONE's policy, we have removed all funding-related information from the manuscript. The Acknowledgment section now includes only general appreciation for institutional and academic support, without reference to specific funding sources.
We note that you have indicated that there are restrictions to data sharing for this study. For studies involving human research participant data or other sensitive data, we encourage authors to share de-identified or anonymized data. However, when data cannot be publicly shared for ethical reasons, we allow authors to make their data sets available upon request. For information on unacceptable data access restrictions, please see http://journals.plos.org/plosone/s/data-availability#loc-unacceptable-data-access-restrictions.<br>Before we proceed with your manuscript, please address the following prompts:<br>a) If there are ethical or legal restrictions on sharing a de-identified data set, please explain them in detail (e.g., data contain potentially identifying or sensitive patient information, data are owned by a third-party organization, etc.) and who has imposed them (e.g., a Research Ethics Committee or Institutional Review Board, etc.). Please also provide contact information for a data access committee, ethics committee, or other institutional body to which data requests may be sent.<br>b) If there are no restrictions, please upload the minimal anonymized data set necessary to replicate your study findings to a stable, public repository and provide us with the relevant URLs, DOIs, or accession numbers. Please<br>see http://www.bmj.com/content/340/bmj.c181.long for guidelines on how to de-identify and prepare clinical	Thank you for this comment.<br><br>Due to the sensitive nature of the data collected in this study, which include household-level socioeconomic and farm management information linked to individual smallholder farmers in Oromia region, public sharing of the full dataset could compromise participant pseudo anonymous. The data were collected under strict confidentiality agreements approved by the Ethics Committee of Haramaya University and in collaboration with the International Potato Center (CIP), both of which require that respondent identities and associated metadata remain confidential.<br>In accordance with institutional and ethical requirements, the dataset cannot be made publicly available. Requests for access may be directed to the corresponding author.

data for publication. For a list of recommended repositories, please see https://journals.plos.org/plosone/s/recommended-repositories. You also have the option of uploading the data as Supporting Information files, but we would recommend depositing data directly to a data repository if possible.<br>Please update your Data Availability statement in the submission form accordingly.	
In the online submission form, you indicated that:<br>"The data will only be available based on a reasonable request."<br>All PLOS journals now require all data underlying the findings described in their manuscript to be freely available to other researchers, either<br>1. In a public repository,<br>2. Within the manuscript itself, or<br>3. Uploaded as supplementary information.<br>This policy applies to all data except where public deposition would breach compliance with the protocol approved by your research ethics board. If your data cannot be made publicly available for ethical or legal reasons (e.g., public availability would compromise patient privacy), please explain your reasons on resubmission and your exemption request will be escalated for approval.<br>7. When completing the data availability statement of the submission form, you indicated that you will make your data available on acceptance. We strongly recommend all authors decide on a data sharing plan before acceptance, as the process can be lengthy and hold up publication timelines. Please note that, though access restrictions are acceptable now, your entire data will need to be made freely accessible if your manuscript is accepted for publication. This policy applies to all data except where public deposition would breach compliance with the protocol approved by your research ethics board. If you are unable to adhere to our open data policy, please kindly revise your statement to explain your reasoning and we will seek the editor's input on an exemption. Please be assured that, once you have provided your new statement, the assessment of your exemption will not hold up the peer review process.	Thank you for this comment.<br><br>See our answer to the previous comment
Please include a separate caption for each figure in your manuscript.	We appreciate the editor's observation. Separate captions have now been added for each figure in the manuscript, clearly describing the content, variables, and data source in line with PLOS ONE formatting guidelines. Each caption appears immediately below its corresponding figure, as required.

If the reviewer comments include a recommendation to cite specific previously published works, please review and evaluate these publications to determine whether they are relevant and should be cited. There is no requirement to cite these works unless the editor has indicated otherwise.	Thank you for this comment.<br><br>No specific citations were recommended by the reviewers. We have therefore confirmed that all references currently included in the manuscript are relevant, accurate, and up to date.
Please review your reference list to ensure that it is complete and correct. If you have cited papers that have been retracted, please include the rationale for doing so in the manuscript text, or remove these references and replace them with relevant current references. Any changes to the reference list should be mentioned in the rebuttal letter that accompanies your revised manuscript. If you need to cite a retracted article, indicate the article's retracted status in the References list and also include a citation and full reference for the retraction notice.	Thank you for this comment.<br><br>We have carefully reviewed the entire reference list to ensure that all citations are complete, accurate, and formatted according to *PLOS ONE* guidelines. All entries were cross-checked for publication status and integrity. None of the cited studies have been retracted, and therefore, no retraction notices were required.<br>In addition, we corrected minor inconsistencies in citation formatting and numbering to align the references precisely with their order of appearance in the manuscript. These adjustments have been made in the revised version.
**# Reviewer 1**	**Response**
I suggest reviewing the title to read "The role of training on smallholder farmers' adoption of orange-fleshed sweet potato in Ethiopia"	Thank you for this comment<br><br>In response, our manuscript title we have revised accordingly "The role of training on smallholder farmers' adoption of orange-fleshed sweet potatoes in Ethiopia."
The manuscript needs editing to enhance language and writing quality	Thank you for this essential comment<br><br>We have conducted a comprehensive, paragraph-by-paragraph revision of the entire manuscript to significantly enhance the language, flow, and sentence structure, resulting in a much stronger and clearer presentation of our findings.
Review the keywords and include "OFSP" and "adoption."	Thank you for this important comment<br><br>We appreciate this valuable suggestion for enhancing the paper's discoverability. We have now incorporated both "OFSP" and "adoption" into the keywords list to accurately reflect the core theme of our study and improve indexing and searchability within academic databases.
The authors should include information on whether the white/cream fleshed sweet potato was cultivated in the studied areas before the introduction of the OFSP.	Thank you for your suggestion.<br><br>As recommended, we have now incorporated an expanded section on the

	experience with OFSP in the study area into the Results and Discussion, with the relevant additions highlighted in yellow within the revised manuscript for ease of reference. It reads as follows:<br><br>"In the study area, farmers were already familiar with cultivating white-fleshed sweet potato varieties before OFSP was introduced. These conventional varieties were mainly grown for household consumption and as a supplementary food source, especially during dry seasons. This prior experience with sweet potato cultivation provided a useful reference for farmers when evaluating OFSP, helping them compare its agronomic performance, taste, and market value with traditional varieties".
In Figure 1, the linkage of training to technology perceptions and behavioral intention is not apparent, as indicated in the text (page 7). Please confirm.	Thank you for your valuable comment.<br><br>We agree that the linkage between the training intervention and technology perceptions/ behavioral intention was not visually explicit in the original figure. We have now revised Figure 1 to clearly illustrate how training influences the TAM and TPB constructs, which in turn shape farmers' behavioral intention.
Minimize abbreviations to the least possible. When there are too many, they distract the reader as they search for meaning. What is PBC (page 11)?	Thank you for your helpful comment.<br><br>We have reduced the number of abbreviations in the manuscript as suggested. Regarding your query, PBC stands for "perceived behavioral control," which is now clearly defined in the revised text.
Describe the scales employed for rating responses in the Materials and Methods section.	Thank you for your comment.<br><br>In this qualitative study, we did not employ any rating scales or quantitative scaling techniques; instead, we relied on open-ended interviews and thematic analysis to capture participants' perspectives. However, we clearly stated this in section 2.2 on study design and experimental procedures, where we reflected upon the scale we used to measure income-related responses.<br><br>"To respect cultural sensitivity around financial matters, farm income satisfaction was measured using a five-

	point Likert scale ranging from 'very dissatisfied' to 'very satisfied."
Was the training mentioned on page 13 about plant or human nutrition?	Thank you for your question.<br><br>The training referred to on page 13 relates specifically to agronomic training, that is, instruction in crop management and good agricultural practices. To make it very clear we have now included the term agronomic
Although concepts and farmers' experiences were assessed qualitatively, if any numerical data was collected, basic statistics such as percentages could be included in the results. Words like some, many, and a few do not sufficiently quantify the population segments.	Thank you for this constructive comment.<br><br>We fully agree that numerical summaries can provide clarity in many research contexts. However, in this study, the purpose of the qualitative component was not to quantify patterns across respondents but to explore the depth of meanings, perceptions, and behavioral mechanisms behind OFSP adoption. Presenting percentages risks suggesting statistical generalization, which is not the foundational approach of qualitative research.<br>Qualitative research seeks to capture the richness and diversity of experiences rather than the frequency with which they occur. Scholars emphasize that quantifying narrative data (e.g., using terms such as "30% said…") can misrepresent qualitative insights by shifting emphasis toward measurement rather than interpretation (Neale et al., 2014). Instead, our use of terms like "many," "several," and "a few" aligns with qualitative reporting conventions intended to preserve conceptual meaning and avoid false precision (Sandelowski, 2001).<br>To maintain methodological integrity, we therefore present themes with contextual explanations rather than numeric summaries. This approach ensures that the farmers' lived experiences, perceptions, and behavioral processes are represented, consistent with the goals of theory-driven qualitative analysis using TPB and TAM frameworks.<br><br>Neale, J., Miller, P., & West, R. (2014). Reporting quantitative information in qualitative research: guidance for authors and reviewers. Addiction, 109(2),175–176. https://doi.org/10.1111/add.12408

	Sandelowski, M. (2001). Real qualitative researchers do not count: The use of numbers in qualitative research. *Research in Nursing & Health*, *24*(3), 230–240. https://doi.org/10.1002/nur.1025
Would the results have differed if the training had also covered food preparation and uses (covering the whole OFSP value chain)? This question may be answered in the Discussion section.	Thank you for this insightful comment.<br><br>The current study intentionally focused on agronomic training as its scope to assess farmers' knowledge and practices related to OFSP production. We acknowledge in the Limitations section that training on food preparation and uses, which addresses the broader OFSP value chain (and consumers in particular), was beyond the study's scope. Future research integrating comprehensive value chain approaches, including post-harvest handling, processing, and consumption, would be valuable to understand the full impact of such training on adoption and utilization.
**# Reviewer 2**	**Response**
Binary Logistic Regression is useful tool to appliy here, If the dependent variable "adoption" is binary (1 = adopted, 0 = not adopted). why was not used here.	Thank you for this valuable comment.<br><br>We agree that binary logistic regression could be an appropriate analytical approach when examining determinants of adoption using quantitative data. However, the present study did not employ logistic regression because this phase of the research is qualitative in design and focuses on understanding how and why agronomic training shapes farmers' perceptions, behavioral intentions, and experiences related to OFSP adoption.<br>Rather than testing statistical associations between variables, the objective here was to explore behavioral mechanisms and context-specific factors using the TAM–TPB framework through in-depth interviews and thematic analysis. As such, a qualitative analytical approach was more appropriate for uncovering rich insights into farmers' motivations, constraints, and attitudes toward OFSP, which cannot be fully captured through regression models.<br><br>Moreover, in this specific study, the primary outcome variable is OFSP yield performance, and not adoption. The current paper focuses on assessing the

	effect of agronomic training on farmers' OFSP production outcomes, specifically yield. Since yield is operationalized as a metric variable, a logistic regression model would not be appropriate for this analysis. Instead, yield-based comparisons and statistical approaches suited to continuous outcomes, i.e. ANOVA, were employed.
Multinomial or Ordered Logistic Regression is also useful tool if you have multiple levels (for example, no adoption, partial adoption, full adoption). It allows differentiation between degrees of adoption behavior.	Thank you for this helpful suggestion. In line with our answer to your previous comment, we agree that multinomial or ordered logistic regression can be appropriate tools when the objective is to statistically differentiate between multiple adoption categories (e.g., no adoption, partial adoption, full adoption).<br>However, in the present manuscript, our analytical focus is not on estimating adoption probabilities or modeling different adoption levels. Instead, this phase of the study specifically evaluates the effect of agronomic training on OFSP yield, with yield as the dependent variable and a continuous outcome. Given this objective and outcome structure, multinomial or ordered logistic regression is not applicable here.<br>Furthermore, the adoption-related insights in this paper were obtained through qualitative analysis, examining how training influenced farmers' perceptions, attitudes, and behavioral processes rather than quantifying adoption levels. As such, qualitative thematic analysis was used to explore mechanisms behind adoption behavior rather than to estimate categorical adoption outcomes.
You should estimate the causal impact of training on adoption by matching trained and untrained farmers who share similar characteristics.	Thank you for the valuable suggestion<br><br>Our results show that the trained and untrained groups were broadly comparable across key characteristics:<br>• Education: 5.4 ± 2.1 years (trained) vs. 5.0 ± 2.3 years (untrained)<br>• Age: 40.8 ± 11.5 years vs. 40.5 ± 10.9 years<br>• Farming experience: 24.9 ± 9.7 years vs. 25.6 ± 10.1 years<br>• Farm size: 0.52 ± 0.19 hectares vs. 0.50 ± 0.21 hectares

- Household composition: Number of young children 1.8 ± 1.0 vs. 2.1 ± 1.2
- Satisfaction with annual household income: 3.6 ± 0.9 vs. 3.5 ± 1.0
- Years of cooperative membership: 12.9 ± 5.8 vs. 11.7 ± 6.1

These similarities demonstrate there was limited variation between groups, making it infeasible to perform matching for causal impact estimation. Random assignment had effectively created balanced groups, thus supporting the causal impact evaluation without the need for additional matching.

Additionally, the methodological choice was primarily constrained by the very small sample size within the experimental groups, which made propensity score matching infeasible. Given this limited sample, further stratified into four subgroups: trained adopters, trained non-adopters, untrained adopters, and untrained non-adopters, applying propensity score matching or similar methods would likely result in insufficient matched pairs and statistically unstable estimates. As Holmes and Olsen (2010) highlight, propensity score methods are highly sensitive to sample size, with very small samples leading to biased or unreliable results. Similarly, Austin (2010) emphasizes that matching procedures require adequate sample sizes to ensure credible estimation and balance across treatment groups. In our study, applying matching would have resulted in a substantial loss of statistical power and compromised the validity of inference.
For these reasons, and consistent with the methodological guidance above, we employed a two-way ANOVA framework to assess the direct impact of training on yield and to examine interactions with adoption status.

Holmes, W., & Olsen, L. (2010). Using propensity scores with small samples. In *Annual meetings of the American Evaluation Association. San Antonio,*

	Texas. https://doi.org/10.13140/2.1.2255.0724.<br><br>Austin P. C. (2010). Statistical criteria for selecting the optimal number of untreated subjects matched to each treated subject when using many-to-one matching on the propensity score. *American journal of epidemiology*, *172*(9), 1092–1097. https://doi.org/10.1093/aje/kwq224
**# Reviewer 3**	**Response**
The abstract effectively summarizes the study but could briefly indicate the analytical approach (qualitative + quantitative integration) to strengthen methodological transparency.	Thank you for this insightful suggestion.<br><br>We agree that briefly clarifying the analytical approach enhances methodological transparency. As recommended, we revised the abstract to reflect the integration of qualitative and quantitative components. Specifically, we have now highlighted that, in addition to qualitative insights derived from pre- and post-intervention interviews, quantitative yield comparisons were incorporated to assess agronomic outcomes. We added a brief statement in the abstract emphasizing the mixed-method nature of the study. Revised text in Abstract (added text highlighted in yellow):<br><br>"…Alongside the qualitative insights, quantitative yield comparisons were incorporated, and the integration of qualitative and quantitative evidence provides valuable insights into how perceptions of benefits, ease of use, and social norms shape adoption behavior…"
The introduction is well-developed; however, consider reducing the number of citations in the early paragraphs to maintain focus.	Thank you for this thoughtful suggestion.<br><br>We appreciate the concern regarding citation density in the introductory section. Our intent in providing multiple references was to establish a strong empirical foundation for three key areas that are central to the study: (1) the global and national significance of vitamin A deficiency, (2) the rationale for OFSP as a biofortified solution, and (3) the existing evidence and research gap regarding adoption challenges. Given that OFSP adoption in Ethiopia is a relatively specialized area, synthesizing diverse literature was important to highlight the breadth and depth of current scholarship and to situate our study clearly within it.

The study objectives could be more sharply stated, preferably in a bullet or numbered list for clarity.	Thank you for this helpful suggestion.<br><br>We agree that clearly stated study objectives strengthen the structure of the introduction. We would like to note that the objectives are already presented in a numbered format.
Please clarify how random assignment to the "trained" and "untrained" groups was ensured, given the purposive nature of the sample.	Thank you for this insightful comment.<br><br>A purposive sample of 65 sweet potato farmers was selected in collaboration with the district administration and Haramaya University Research Institute. After eligible farmers were identified based on predefined inclusion criteria namely experience with sweet potato production and willingness to participate farmers were randomly assigned to "trained" (treatment) and "not-trained" (control) groups within this purposive sample. This random allocation ensured that each participant had an equal chance of receiving the training, allowing for unbiased evaluation of the training's effect on adoption determinants, intention, and yield outcomes.<br><br>In summary, while the overall sample was purposively chosen to ensure contextual relevance, the intervention itself, the training, was randomly assigned to subgroups of participants.<br><br>This mixed approach purposive sampling for eligibility followed by random assignment for treatment allocation is a well-established method in field-based behavioral and agricultural research (see Onwuegbuzie et al., 2015; Glennerster & Takavarasha, 2013), allowing both representativeness and experimental control.<br>Glennerster, R., & Takavarasha, K. (2013). Running randomized evaluations: A practical guide. In running randomized evaluations. Princeton University Press.<br><br>Onwuegbuzie, A., & Collins, K. (2015). A typology of mixed methods sampling designs in social science research. *The Qualitative Report*. https://doi.org/10.46743/2160-3715/2007.1638

In section 2.4, the description of ANOVA results should include assumptions testing (normality, homogeneity) or a statement confirming their validation.	Thank you for this helpful comment. We have now included a statement in Section 2.4 confirming that the assumptions of normality and homogeneity were tested and met prior to conducting the ANOVA. The Shapiro–Wilk and Kolmogorov–Smirnov tests indicated normality ($p > 0.05$), and Levene's test confirmed homogeneity of variances ($p > 0.05$). The revised text appears at the end of Phase 2 in Section 2.4 (added text highlighted in yellow):<br><br>"Before conducting the ANOVA, key assumptions were verified. Normality of residuals was assessed using the Shapiro–Wilk and Kolmogorov–Smirnov tests, which indicated no significant deviations from normality ($p > 0.05$). Homogeneity of variances was tested using Levene's test, which confirmed equal variance across groups ($p > 0.05$). These results validated the use of ANOVA for the analysis. Therefore, the test results presented in the results section are based on data that met the underlying statistical assumptions …."
The qualitative findings are rich; however, the use of direct quotations from farmers could be reduced slightly or summarized to improve readability.	Thank you for this comment.<br><br>In response, we have carefully reduced quotations to enhance readability while preserving the richness and authenticity of farmers' voices. Only the most illustrative and representative quotes have been retained to support key themes. This revision ensures a stronger balance between analytical interpretation and empirical illustration in the qualitative sections.
It would be helpful to include a short table summarizing key pre- and post-training differences in TAM/TPB constructs.	We thank the reviewer for this suggestion.<br><br>In response, we have added a new summary table (Table 3) that highlights the key differences in farmers' perceptions before and after the agronomic training across the Technology Acceptance Model (TAM) and Theory of Planned Behavior (TPB) constructs. This addition enhances clarity and provides a concise overview of how training influenced behavioral determinants, complementing the qualitative discussion in Sections 3.2.2–3.2.6 and 3.3.1.

Ensure all figures (e.g., Figure 1, Figure 2) and tables (Tables 1–3) are properly formatted and labeled according to journal style guidelines. Some figure insert markers remain (“[INSERT FIGURE 1]”).	Thank you for this comment.<br><br>All figure and table insert markers (e.g., “[INSERT FIGURE 1]”) have been removed. Each figure and table is now properly numbered, formatted, and labeled according to the PLOS ONE journal style and requirements.
The ethics statement is clear, but the Data Availability section should conform fully to journal requirements.	We thank the reviewer for this observation.<br><br>The Data Availability statement has been revised to fully comply with PLOS ONE requirements. Due to the sensitive nature of the household and farmer-level data and the confidentiality agreements with Haramaya University and the International Potato Center (CIP), the dataset cannot be made publicly available. Requests for access may be directed to the corresponding author.
Minor grammatical and typographical errors (e.g., missing spaces before parentheses, inconsistent use of “non-adopters”) should be corrected.	Thank you for this comment.<br><br>These have been carefully reviewed and corrected throughout the manuscript. We have ensured consistent use of terms such as “non-adopters” and addressed missing spaces before parentheses, as well as any other formatting inconsistencies. The manuscript has been thoroughly proofread to improve clarity and readability.
Ensure consistency in referencing style (check capitalization and journal formatting).	Thank you for the suggestion.<br><br>The references have been thoroughly reviewed and corrected for consistency in capitalization, journal title formatting, and overall referencing style. We have ensured adherence to the journal’s referencing guidelines throughout the manuscript.
The conclusion is strong; you might add one sentence on how the findings could inform broader biofortification scaling strategies beyond Ethiopia.	We thank the reviewer for this insightful suggestion.<br><br>A new sentence has been added to the conclusion (highlighted in yellow) to emphasize how the study’s insights extend beyond Ethiopia. Specifically, the revised text clarifies that the behaviorally informed, training-based approach identified here can be adapted to similar smallholder farming contexts in sub-Saharan Africa and other low-income regions, thereby informing broader biofortification scaling strategies.

	"Moreover, the findings contribute to the broader contexts of biofortification by showing how behaviorally informed, training-based interventions can be adapted to similar smallholder contexts across sub-Saharan Africa and other low-income regions, thus supporting the scaling of nutrition-sensitive agriculture beyond Ethiopia".
