# Supplementary material for: The role of training on smallholder farmers’ adoption of orange-fleshed sweet potato in Ethiopia
Source: PLoS One. 2026 Jan 20;21(1):e0340709. doi: 10.1371/journal.pone.0340709 (PMC12818620; doi:10.1371/journal.pone.0340709)
Supplement: Attachment — Submitted filename: PONE-D-25-37573.docx [file pone.0340709.s004.docx]

**The role of training on smallholder farmers' adoption of biofortified crops: evidence from orange-fleshed sweet potatoes in Ethiopia**

This manuscript titled *“The role of training on smallholder farmers' adoption of biofortified crops: evidence from orange-fleshed sweet potatoes in Ethiopia”* presents an insightful and well-structured study that combines the Theory of Planned Behavior (TPB) and the Technology Acceptance Model (TAM) within a qualitative experimental framework. The topic is timely and relevant to agricultural extension, biofortification and smallholder adoption behavior in Sub-Saharan Africa. The manuscript is clearly written, methodologically sound and contributes to the understanding of how training interventions influence both behavioral and agronomic outcomes. I commend the authors for their rigorous fieldwork and the integration of social-psychological models into agricultural adoption research.

However, before acceptance, I suggest **minor revisions** to improve clarity, transparency and flow.

1. **Abstract clarity:**
   - The abstract effectively summarizes the study but could briefly indicate the analytical approach (qualitative + quantitative integration) to strengthen methodological transparency.
2. **Introduction:**
   - The introduction is well-developed; however, consider reducing the number of citations in the early paragraphs to maintain focus.
   - The study objectives could be more sharply stated, preferably in a bullet or numbered list for clarity.
3. **Methodology:**
   - Please clarify how random assignment to the “trained” and “untrained” groups was ensured, given the purposive nature of the sample.
   - In section 2.4, the description of ANOVA results should include assumptions testing (normality, homogeneity) or a statement confirming their validation.
4. **Results and Discussion:**
   - The qualitative findings are rich; however, the use of direct quotations from farmers could be reduced slightly or summarized to improve readability.
   - It would be helpful to include a short table summarizing key pre- and post-training differences in TAM/TPB constructs.
5. **Figures and Tables:**
   - Ensure all figures (e.g., Figure 1, Figure 2) and tables (Tables 1–3) are properly formatted and labeled according to journal style guidelines. Some figure insert markers remain (“[INSERT FIGURE 1]”).
6. **Ethics and Data Availability:**
   - The ethics statement is clear, but the Data Availability section should conform fully to journal requirements.
7. **Language and formatting:**
   - Minor grammatical and typographical errors (e.g., missing spaces before parentheses, inconsistent use of “non-adopters”) should be corrected.
   - Ensure consistency in referencing style (check capitalization and journal formatting).
8. **Conclusions:**
   - The conclusion is strong; you might add one sentence on how the findings could inform broader biofortification scaling strategies beyond Ethiopia.

**Recommendation**

**Decision:** Minor revision
The study is methodologically robust, original and of potential high impact for agricultural policy and development practice. Addressing the above points will enhance clarity and ensure alignment with *PLOS ONE* editorial standards.
